# Understanding Engagement in Dementia Through Behavior. The Ethographic and Laban-Inspired Coding System of Engagement (ELICSE) and the Evidence-Based Model of Engagement-Related Behavior (EMODEB)

**DOI:** 10.3389/fpsyg.2018.00690

**Published:** 2018-05-24

**Authors:** Giulia Perugia, Roos van Berkel, Marta Díaz-Boladeras, Andreu Català-Mallofré, Matthias Rauterberg, Emilia Barakova

**Affiliations:** ^1^Designed Intelligence, Industrial Design, Eindhoven University of Technology, Eindhoven, Netherlands; ^2^Technical Research Center for Dependency Care and Autonomous Living, Automatic Control Department, Technical University of Catalonia, Vilanova i la Geltrú, Spain

**Keywords:** nonverbal behavior, body movement, Laban Movement Analysis, ethogram, coding system, dementia, engagement, structural equation modeling

## Abstract

Engagement in activities is of crucial importance for people with dementia. State of the art assessment techniques rely exclusively on behavior observation to measure engagement in dementia. These techniques are either too general to grasp how engagement is naturally expressed through behavior or too complex to be traced back to an overall engagement state. We carried out a longitudinal study to develop a coding system of engagement-related behavior that could tackle these issues and to create an evidence-based model of engagement to make meaning of such a coding system. Fourteen elderlies with mild to moderate dementia took part in the study. They were involved in two activities: a game-based cognitive stimulation and a robot-based free play. The coding system was developed with a mixed approach: ethographic and Laban-inspired. First, we developed two ethograms to describe the behavior of participants in the two activities in detail. Then, we used Laban Movement Analysis (LMA) to identify a common structure to the behaviors in the two ethograms and unify them in a unique coding system. The inter-rater reliability (IRR) of the coding system proved to be excellent for cognitive games (kappa = 0.78) and very good for robot play (kappa = 0.74). From the scoring of the videos, we developed an evidence-based model of engagement. This was based on the most frequent patterns of body part organization (i.e., the way body parts are connected in movement) observed during activities. Each pattern was given a meaning in terms of engagement by making reference to the literature. The model was tested using structural equation modeling (SEM). It achieved an excellent goodness of fit and all the hypothesized relations between variables were significant. We called the coding system that we developed the Ethographic and Laban-Inspired Coding System of Engagement (ELICSE) and the model the Evidence-based Model of Engagement-related Behavior (EMODEB). To the best of our knowledge, the ELICSE and the EMODEB constitute the first formalization of engagement-related behavior for dementia that describes how behavior unfolds over time and what it means in terms of engagement.

## Introduction

Engagement in activities is of crucial importance for people with dementia (Kolanowski et al., 2006; Brooker et al., 2007). Indeed, a growing body of research has found that participation in activities is associated with augmented self-efficacy and self-esteem in dementia (Benveniste et al., 2012) and is deemed useful to improve social bonding (Wada and Shibata, 2008) and mood (Moyle et al., 2013) and to reduce loneliness (Robinson et al., 2013), challenging behaviors (Mordoch et al., 2013), and medication consumption (Moyle et al., 2015).

At present, the benefits of participation in activities are predominantly measured with regard to their long-term clinical outcomes. However, there is an intermediate step between participation in activities and clinical gain that the majority of literature on dementia overlooks: when activities are meaningful, they have a higher clinical resonance (Cohen-Mansfield et al., 2010). A systematic assessment of engagement through behavior could be greatly beneficial to measure the meaningfulness and effectiveness of activities for the person with dementia and could be used as a complement to the assessment of clinical benefits. We define engagement as the psychological state of well-being, enjoyment and active involvement that is triggered by meaningful activities and causes people with dementia to be absorbed by the activity, more energetic and in a more positive mood (Perugia et al., 2017c).

Engagement is usually measured using self-reports (Csikszentmihalyi and Larson, 1987; IJsselsteijn et al., 2008). However, as dementia progresses, self-reports become an unfeasible form of assessment, since retrieval, reporting, and ranking of relevant information, especially if located in the past, gets significantly compromised. This is the reason why almost all assessment techniques of engagement for dementia rely on behavior observation. There are three ways to measure engagement through behavior: observational rating scales, ethograms, and coding schemes. An observational rating scale is a collection of items measured on a Likert scale and operationalized through behavior. An ethogram is the complete inventory of species-related behavior. Last, a coding scheme is an excerpt of an ethogram aimed at answering specific research questions. In the context of engagement assessment for dementia, observational rating scales are too general to grasp how engagement manifests itself through behavior and unfolds over time. Ethograms produce a complete yet segmented picture of engagement difficult to report to an overall state of engagement. Coding schemes are mostly developed by cherry-picking the target behaviors without the preventive development of an ethogram.

In order to solve this problem, we carried out a longitudinal study involving people with dementia in two very diverse activities: a game-based cognitive stimulation and a robot-based free play. This study had a twofold objective: (i) develop a reliable coding system of engagement-related behavior that saves the complexity of an ethogram but can be used across activities and (ii) create a model of engagement that describes how engagement-related behavior unfolds and how it can be interpreted in engagement terms.

To pursue the first objective, we employed a mixed approach: ethographic and Laban-inspired. First, we observed people with dementia during the two activities and developed two ethograms, one per activity, to describe their behavior. Second, we used Laban Movement Analysis (LMA; Laban, 1966) to identify a common structure to the behaviors in the two ethograms and unify them in a unique coding system workable across activities. In this phase, we used the category shape of LMA, which formalizes how the body changes shape to respond to inner motives and to the environment. Moreover, we tested the inter-rater reliability (IRR) of the coding system to ensure that it was reliable across coders.

To pursue the second objective, we first scored all the videos collected during the study. Second, we identified patterns of behaviors recurring across sessions. To do this, we used the category body of LMA in the formalization of body part organization. Body part organization describes how body parts are connected in movement. As a last step, we summarized the patterns of behaviors into a model and tested the goodness of fit of the model using structural equation modeling (SEM). Each pattern of behavior was given a meaning in terms of engagement by making reference to the literature.

## Related work

### Observational rating scales

Early measurements of engagement came in the form of observational rating scales. Observational rating scales are ordinal Likert-type scales that measure engagement through behavior. The most widely used in the field of gerontology is the Observational Measurement of Engagement (OME) developed by Cohen-Mansfield et al. (2009). In the OME, engagement is defined as “the act of being involved or occupied with a stimulus” and is measured across four dimensions: *duration* (time in seconds that the person with dementia is involved with the stimulus), *attention* (attentional allocation toward the stimulus measured on a 4-point Likert-scale), *attitude* (affective stance toward the stimulus measured on a 7-point Likert scale), and *refusal* (acceptance or rejection of the stimulus). Another broadly employed observational scale of engagement is the Menorah Park Engagement Scale (MPES), which has been developed by Judge et al. (2000) to assess engagement in people with dementia involved in Montessori-based interventions. In the MPES, engagement is defined as “motor or verbal behavior exhibited in response to the activity” and is assessed along a single item, engagement, that can take four values: *non-engagement* (no motor or verbal behavior in response to the activity, e.g., stare into space, look away from the activity), *self-engagement* (self-directed motor and verbal behavior in response to the activity, e.g., hand-wringing), *passive engagement* (passive motor and verbal behavior directed toward the activity, e.g., looking toward the activity, listening) and *constructive engagement* (proactive motor and verbal behavior directed toward the activity, e.g., manipulating objects, talking). A last observational scale that is widely used in dementia is the Observed Emotion Rating Scale (OERS; Lawton et al., 1996). It does not directly measure engagement but has often been used in concert with the OME and MPES to assess the emotional state of people with dementia during activities (Moyle et al., 2013; Perugia et al., 2017b). The OERS measures the intensity or duration of five affective states along a 5-point Likert-scale: *pleasure, anxiety/fear, anger, sadness*, and *general alertness*.

Observational rating scales are very useful tools to get a broad idea of the engagement state of the person with dementia during activities. However, they can grasp engagement only at a global level. Indeed, they do not get into the detail of how behavior naturally occurs and unfolds. They collect a general idea of engagement which is drawn from the occurrence of certain behaviors.

### Coding schemes

A different approach toward measuring engagement is for instance adopted in the field of socially interactive robotics (SIR) where the study of interactions between humans and social robots is of crucial importance (Pino et al., 2015). Socially interactive robots are robots that engage socially with humans for the sake of social interaction itself (Feil-Seifer and Mataric, 2011). In the context of SIR, a considerable effort has been done to understand how people with dementia interact with social robots and how such an interaction could have a therapeutic value (Bemelmans et al., 2012; Valentí Soler et al., 2015; Rouaix et al., 2017). To understand the meaningfulness of the interactions that social robots promote, researchers have compiled repertoires of behaviors and used them to annotate videos. Just to make few examples, Takayanagi et al. (2014) used a time sampling method to compare the effects of the social robot PARO (the arctic seal robot) to those of a stuffed animal (a lion) in people with mild/moderate and severe dementia. They divided videos into units of 10 s and at each interval scored whether the observed person *talked* (to PARO/lion, to the staff, to him/herself or to nobody), *touched* or *stroked* (PARO/lion), and had *positive, neutral* or *negative facial expression*. Šabanović et al. (2013) explored the behavior behind PARO's therapeutic success by coding *visual engagement* (look at the robot), *verbal engagement* (speak, sing, vocalizations toward the robot), and *physical engagement* (pet, hit, hold, kiss, take/offer PARO). Wada et al. (2010) tested the effectiveness of a manual for the use of PARO with people with dementia by scoring engagement on a coding sheet that comprised the classes: *emotional expression* (laugh, smile, no expression, hate), *gaze* (PARO, staff, user, others), *talk* (PARO, staff, user, others), and *type of interactions with PARO* (give, stroke, hold, other). Coding schemes have been employed also in other contexts. For instance, to assess engagement in multi-sensory and motor stimulation programs. Cruz et al. (2011) assessed engagement during these types of interventions using a coding scheme composed of the following categories: *engagement in the task, interactions with objects, verbal communication, smiling, laughing, nodding the head*, and *closed eyes*.

The just described coding schemes provide a deeper understanding of behavior compared to observational scales. However, they grasp only some characteristics of behavior. Indeed, instead of considering behavior in its natural flow, they fragment it to pick up only the desired pieces of information. In these cases, since the fragmentation of behavior is not performed in a systematic way, it results in a cherry-picking of behaviors based on their perceived meaningfulness. Ideally, a researcher should first develop a complete inventory of behaviors (ethograms) and then focus on a portion of it (coding schemes) based on research questions. However, such practice is not reported in these studies.

### Ethograms

Ethology is the discipline that studies animal behavior from a biological perspective. As a discipline, Ethology faces nearly the same constraint as gerontology for dementia: the inaccessibility of mental experiences (Troisi, 1999). To address this issue, Ethology has elaborated a very distinctive and powerful method of analysis which is rooted in direct observation, rigorous description and objective analysis of behavior, the ethogram.

The words ethogram and coding scheme are often used as synonyms in the literature. Some authors use the word ethogram as a synonym for coding scheme (Cruz et al., 2011), others use the word ethogram to designate a more thorough description and analysis of behavior that stems from field observation and incorporates a good deal of complexity (Mabire et al., 2016). In Ethology, the ethogram is the complete list of actions that a particular species performs, while the coding scheme is a portion of an ethogram aimed at answering specific research questions.

Recently, several ethograms have been developed to assess engagement in dementia. Olsen et al. (2016) gauged engagement in people with dementia involved in Animal Assisted Activities (AAA) using an ethogram that comprised the following behaviors: *conversation* (unspecified target), *look at* (other people, the dog activity, other things), *touch* (people, dog), *smile, or laugh at* (dog, other things), *sing/dance/clapping hands, stereotyped behavior, wandering around, agitated behavior, yawn*, and *sigh, no response, asleep, leaving the room, off camera*. Jøranson et al. (2016) studied the behaviors of people with dementia involved in interactions with PARO and grouped them in: *conversation* with or without PARO, *observe* (PARO, other participant/activity leader, other things in the room), *smile/laughter* (PARO, other participant/activity leader), *physical contact* with PARO, *active* with PARO, *singing/whistling, clapping/humming/dancing, napping, walking around, repetitive movement, time out of recording, physical contact* (with participant/activity leader), *signs of discomfort, leaving the group, no response to contact*. Perhaps one of the most complete ethograms of engagement built for dementia is the Video-Coding Incorporating Observed Emotions (VC-IOE; Jones et al., 2015) The VC-IOE was compiled to assess the engagement of people with dementia with mobile telepresence and companion robots. It has six dimensions: *facial emotional response* (the OERS items: pleasure, anxiety/fear, anger, sadness, general alertness, none), *verbal engagement* (positive verbal engagement with stimulus, positive verbal engagement with facilitator, negative verbal engagement, no verbal engagement, missing), *visual alertness/engagement* (visually engaged with stimulus, visually engaged with facilitator/others, no visual engagement, missing visual), *behavioral engagement* (positive behavioral engagement, negative behavioral engagement, no behavioral engagement, missing behavior), *collective engagement* (using stimulus for collective engagement, no evidence of collective engagement), and *agitation* (based on Cohen-Mansfield Agitation Inventory—CMAI: evidence of agitation and no evidence of agitation; Koss et al., 1997).

The ethograms that we have described are optimal to study behavior in its complexity as it naturally occurs and flows. However, they produce a measurement of engagement that is segmented into many small pieces of information that cannot be traced back to an overall engagement state. For this reason, we decided to employ a mixed approach to develop a coding system: ethographic and Laban-inspired. First, we observed people with dementia involved in two very different activities and developed two ethograms to describe their behavior in the two contexts in a detailed way. At this level, we kept behaviors to a very fine granularity. Second, we used LMA to identify a common organizational structure to the behaviors in the two ethograms and unify them in a unique coding system viable for both activities^1^.

### Laban movement analysis

LMA is a holistic framework that provides a vocabulary to describe, interpret and generate movement (Bartenieff and Lewis, 1980). It is organized into four main categories: body, space, effort, and shape (Hackney, 2002). The category *body* defines specific body parts (in terms of elements in the body structure) and how these body parts are connected in movement (Maletic, 1987). The orchestration of body parts (also called *body part organization*) can be *successive* (adjacent body parts move one after the other), *sequential* (non-adjacent body parts move one after the other), and *simultaneous* (all active body parts move together at the same time). The category *space* describes the specific direction of a movement with the center of the body as a reference point. The aim of this category is mapping the 3-dimensional structure of the body in relation to the 3-dimensional environment. The category *effort* regards the qualities of movement, how a movement is performed. The movement has four qualities: *flow* (ongoingness), *weight* (relating to power and gravity), *space* (focus), and *time* (change in speed) (Bradley, 2008). The category *shape* describes “attitudes toward the environment that are expressed in the way the body changes form” (Wile and Cook, 2010). There are three distinctions in the category shape, also referred to as *modes of shape change*: *shape flow* (changes in shape in relation to the self), *directional shape* (goal-oriented changes of the body shape in relation to the others and the environment), and *shaping* (molding and carving of the body in interaction with the others and the environment).

In the past, LMA has been used in numerous studies. For instance, to create and describe choreographies (Preston-Dunlop, 1995), recognize emotions in dance movement (Camurri et al., 2003), increase movement efficiency for factory workers (Lamb, 1965), develop “choreographies of interaction” for design activities (Weerdesteijn et al., 2005), communicate emotions and mental states to robots (Lourens et al., 2010) and evoke and intensify the perception of emotions (Shafir et al., 2016).

In order to organize the ethograms in a unique coding system, we focused on the category shape and, in particular, on the modes of shape change. This was for three reasons. First, the category shape captures the way the body changes shape in relation to the self and the environment, and, in general, the behaviors in the ethograms mostly expressed a direction of the body toward the environment (other participants, facilitator, and game) that had a neutral, positive or negative affective nuance. Second, the modes of shape change conceive the body in its entirety and describe changes in its form as whole-body dynamics. This gave us the possibility to describe a large variety of body configurations by combining behaviors belonging to different body parts. Third, as the modes of shape change describe whole-body dynamics motivated by inner attitudes and by the environment, they were particularly suited to associate an engagement meaning to the different body configurations described^2^.

### Frameworks of engagement

At present, there is just one model of engagement developed for people with dementia, the Comprehensive Process Model of Engagement (Cohen-Mansfield et al., 2011). It describes a series of factors that influence engagement (measured with the OME) in people with dementia: *environmental attributes* (e.g., background noise, lighting, sound, number of persons in proximity) *stimuli attributes* (e.g., human social stimuli, simulated social stimuli, inanimate social stimuli) and *personal attributes* (e.g., gender, age, marital status, medication intake). As the experience of engagement is very difficult to study in people with dementia, very little is known on its characteristics and components. To draw a thorough framework of engagement for dementia, we must step into other domains and understand whether renowned models of user engagement are applicable to dementia.

Attfield et al. (2011) described user engagement as the “emotional, cognitive and behavioral connection that exists, at any point in time, and possibly over time, between a user and a resource.” Such connection is described by a series of characteristics: *focused attention, positive affect, aesthetics* (i.e., the sensory and visual appeal of an interface), *endurability* (i.e., the likelihood of remembering an experience), *novelty* (i.e., the surprise effect provoked by a new experience), *richness and control* (i.e., the variety and complexity of thoughts, actions and perceptions evoked by the activity), *reputation-trust-expectation* and *user context* (i.e., the motivation, incentives, and benefits that users get from engagement). Some of these characteristics—namely endurability, novelty, richness and control—are difficult to study in dementia since they suppose preserved cognitive skills. Other characteristics—aesthetics and reputation-trust-expectation—are features of the technology influencing engagement, rather than elements composing it. Three elements of this framework might be transferred to the context of dementia: focused attention, positive affect and user context. Attentional and emotional involvement are unanimously considered the fundamentals of user engagement (Cohen-Mansfield et al., 2009; Peters et al., 2009). User characteristics are called *personal attributes* by Cohen-Mansfield et al. (2011) and are proved to affect engagement in dementia. Indeed, Perugia et al. (2017b) found out that motivational disorders, such as apathy and depression, negatively affect engagement in dementia.

When engagement is studied in the context of HRI, things change. Castellano et al. (2009) involved children in a chess play with the robot iCat. They observed that, in such a context, engagement got influenced both by the *task* that the user had to carry out and by the *social* interaction with the agent. In general, the framework of Castellano and colleagues is applicable to dementia. Indeed, playful activities are usually carried out in groups in nursing homes. As a matter of fact, Perugia et al. (2017a) applied thematic analysis to an inventory of behaviors displayed by people with dementia during playful activities and identified three main themes overlapping with those just described: *attention* (task-centered engagement), *rapport* (social interaction), and *affect*.

In the literature of user engagement, engagement is regarded as a process composed of a number of stages. Sidner et al. (2005) defined engagement as “the *process* by which individuals in an interaction *start, maintain*, and *end* their perceived connection to one another.” O'Brien and Toms (2008) identified four phases of engagement: *point of engagement, sustained engagement, disengagement*, and *re-engagement*. The conception of engagement as a process with a beginning, a development and an end can be easily reported to the context of dementia, especially if we are able to create a systematic description of the progression of engagement-related behavior over time.

A last feature of engagement to mention is its intensity. Brown and Cairns (2004) observed three levels of the immersion in the game experience: *engagement* (the gamer invests time, effort, and attention), *engrossment* (the gamer's emotions are directly affected by the game), and *total immersion* (the gamer is cut off from reality, all that matters is the game). The first two levels—engagement and engrossment—can be transposed to the context of dementia as they can be gauged with objective measures (e.g., behavior, physiology). The latter—total immersion—cannot. Indeed, it must be assessed with subjective measures (e.g., self-reports) and it is related to a sense of *detachment from reality* and *loss of spatial and temporal reference points* that constitutes the normal condition of people with dementia.

To summarize, according to the literature, engagement is composed by focused attention (or task-engagement), social interaction (or rapport), and affect. It is a process that has a start (or point of engagement), a development (or sustained engagement), and an end (disengagement) and has different levels of intensity: engagement, engrossment, and total immersion. Within this paper, we present an evidence-based model of engagement-related behavior (EMODEB) that tries to report all these features of engagement to the context of dementia.

## Materials and methods

### Participants

Fourteen elderlies ranging in age from 69 to 92 years (*M*: 83.93, *SD*: 7.28) with a diagnosis of dementia took part in the study. Dementia severity was assessed with the Reisberg Global Deterioration Scale (scores of 4 or 5; Reisberg et al., 1993) and the *Mini-Examen Cognoscitivo* (MEC, the Spanish version of the Mini-Mental State Examination, scores between 10 and 23; Vinyoles Bargalló et al., 2002). Inclusion criteria for the participation in the study were a diagnosis of mild to moderate dementia and the informed consent of both the participants and their legal guardians. Exclusion criteria were severe dementia, acute visual impairment, bed-ridden condition, reduced motility in the upper limbs, Parkinson's disease or Parkinson's disease dementia and strong hallucinatory or delusional states.

Participants meeting the inclusion criteria were randomly coupled and participated in the study in pairs (seven couples). The participants in the couples did not know each other prior to the start of the research. The coupling of participants was aimed at preserving the ecological validity of the study by creating a context as close as possible to that of a real-life activity, which is usually group-based.

The decision of excluding participants with severe dementia from this study was dictated by the need to identify behaviors strictly related to engagement. In severe dementia, ambiguous behaviors might appear during activities. For instance, severe dementia might cause participants to sleep during activities. In engagement terms, sleeping represents a lack of interest toward the activity. However, in the case of severe dementia, it might be as well due to the severity of the medical condition.

Another deterrent for the inclusion of people with severe dementia in this study was the sparsity of engagement-related behaviors displayed by people with severe dementia as compared to people with mild and moderate dementia during a pilot study. We assumed that the inventory of engagement-related behaviors would have been more complete if we had focused on participants with mild and moderate dementia. Moreover, we theorized that it would have constituted a superset of an inventory of engagement-related behaviors compiled with persons with severe dementia.

### Design

The study followed a repeated measures design with two conditions: a game-based cognitive stimulation and a robot-based free play. Each activity was presented in a different session and was repeated three times within the study. As a result, the study was composed of six sessions, three of game-based cognitive stimulation and three of robot-based free play. The two activities were presented in alternated order across sessions. Game-based cognitive stimulation and robot-based free play were presented to participants every other week starting from game-based cognitive stimulation (see Table 1 for an overview of the study design).

**Table 1:** Overview of the study design.

**First session**	**Second session**	**Third session**	**Fourth session**	**Fifth session**	**Sixth session**
Jigsaw puzzle 1		Dominoes		Shape puzzle 1	
Jigsaw puzzle 2		Jigsaw puzzle 1		Shape puzzle 2	
Jigsaw puzzle 3		Jigsaw puzzle 2		Shape puzzle 3	
Shape puzzle 1	Play with Pleo	Jigsaw puzzle 3	Play with Pleo	Dominoes	Play with Pleo
Shape puzzle 2		Shape puzzle 1		Jigsaw puzzle 1	
Shape puzzle 3		Shape puzzle 2		Jigsaw puzzle 2	
Dominoes		Shape puzzle 3		Jigsaw puzzle 3	

All sessions of activities were conducted by a clinician working in the nursing homes (i.e., the psychologist or the social educator of the care facility) at the presence of an experimenter (i.e., a researcher from the university). The pairing of facilitators with the couples was random, and the same clinician followed the same couples across all the sessions. The experimenter was present during sessions to ensure the timely execution of activities and to monitor the functioning of the equipment. In order for his/her presence not to be disruptive of the behavior of participants, the experimenter took part in the activities of the nursing homes for 1 month prior to the start of the study.

### Activities

In the game-based cognitive stimulation, participants were asked to collaboratively complete three types of board games: jigsaw puzzles, shape puzzles and a game with dominoes. The jigsaw puzzles and the shape puzzles to complete were three. They were presented in a progressive order of difficulty, from the easiest to the most difficult across sessions (Table 1). The challenge level of jigsaw puzzles was customized according to the cognitive level of the participants in the couples. The right level of challenge was determined in a pilot study. Couples with one or both participants with mild dementia completed two 6-piece puzzles and one 9-piece puzzle. Couples with both participants with moderate dementia completed two 4-piece puzzles and one 6-piece puzzle. The order of presentation of board games was randomized using a Latin squares technique and was always different across sessions.

**Figure 1:**
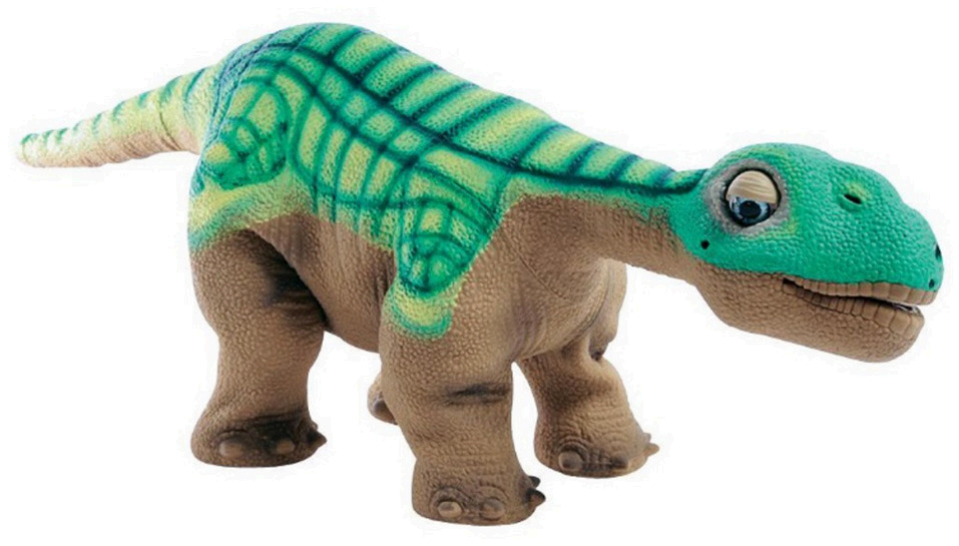
The animatronic pet robot, Pleo.

In the robot-based free play, participants interacted with the animatronic pet robot Pleo (http://www.pleoworld.com/pleo_rb/eng/lifeform.php). Pleo is a robotic dinosaur developed by UGOBE which acts as a living pet (Figure 1). It has an array of sensors that allow it to make sense of the surrounding environment and interact with people. For instance, touch sensors to discriminate among different types of touch, microphones to perceive sound and orientate toward it, ground foot sensors to detect surfaces, a camera-based vision system to detect light and navigate and an internal clock to recognize the time to get up, eat or sleep. Pleo is also able to display its internal states (e.g., hunger, sleep) and moods (e.g., happy, scared). During sessions, participants were left free to interact with the robot spontaneously. The facilitators were given a script with a list of activities that Pleo could support (e.g., feed Pleo, make Pleo sleep), so that they could prompt further interaction in case of a deadlock.

The two activities were chosen for two reasons: they involved different skills (game-based cognitive stimulation: cognitive skills, robot-based free play: affective and social skills) and the artifacts used in the two activities had very different affordances (Norman, 1999). As a consequence, the two activities were likely to prompt different engagement states and different behavioral expressions of such engagement states. The fact that the two activities were likely to elicit different types of engagement was of substantial importance. Indeed, this study was aimed at developing a coding system of engagement-related behavior applicable to diverse activities.

### Setting

The study took place in two nursing homes in rooms that were usually allocated to recreational activities. A rectangular table was placed on one side of the room, and two hand-held cameras were arranged on top of mini-tripods and positioned one in front and one on the side of participants. The frontal camera was positioned on a small table, the lateral camera was either hidden on a library shelf or positioned on a desk.

During activities, the participants sat on the same side of the table. The facilitator always stood between the participants. The central positioning of the facilitator was meant not to influence the engagement state of the participants. Indeed, in a previous pilot study, we noticed that, when the facilitator spent more time closer to one of the participants, this had a negative influence on the flow of the session.

### Instruments and measures

#### Video recording

The cameras were switched on by the experimenter as participants reached the room and were turned off by the same when they left. Before the start of each session, facilitator and experimenter ran a habituation phase. They conversed shortly with participants about their week to get them accustomed to the experimental setting and comfortable with the situation of data collection. Albeit the presence of cameras might be thought of as a factor that could affect participants' behavior, we noticed that participants forgot about the cameras as the activity started.

To develop the ethograms, we used the video footage of the frontal camera. The lateral camera was used as a back-up in case the frontal camera did not work, or objects occluded the full visibility of behaviors. The original videos collected from the frontal camera were cut from the beginning of the activity to the end of the activity. Hence, the habituation phase was not considered in the development of the coding system and was also left out of the scoring. We considered the moment when the facilitator placed the first board game or Pleo on the table in front of the participants as the *beginning of the activity* and the moment when s/he removed the last board game or Pleo from the table as the *end of the activity*. The database of videos was composed of 42 sessions of play of the duration of 20–25 min (~17.5 h of video footage).

The database of videos was split in two parts. Thirty videos were used to develop the ethograms and structure them in a coding system (15 videos per activity type). Twelve videos (6 videos per activity type) were used to test the IRR of the coding system.

#### Building the coding system

The coding system of engagement-related behavior was built in 18 months by a multidisciplinary research team which included a certified movement analyst (CMA). The development of the coding system consisted of two phases: *description* and *structuring*. In the descriptive phase, we adopted an ethographic approach similar to that of Olsen et al. (2016) and Jøranson et al. (2016) and developed two ethograms, one per activity. In the structuring phase, we employed LMA to sort out the complexity of the two ethograms. This enabled us to identify commonalities among the behaviors displayed in the two activities and interpret each behavior in engagement terms.

##### Methodological note on the development of the coding system

The decision of building two ethograms, one per activity, instead of just one, had three motives. First, although the ethogram is operationalized as a catalog of species-related behavior in Ethology, ethologists acknowledge the role of context in shaping behavior. As a matter of fact, animal behavior broadly changes when studied in captivity and in the wild. Second, when behavior is kept to a very fine granularity, it is considerably influenced by the affordances of the objects populating the scene under study and by their use. Albeit stroking a robot and holding the pieces of a jigsaw puzzle can both be conceived as manipulations of a game, they are motivated by the specific affordances of the artifacts in use. Third, as the two activities under study had very different scopes of action (cognitive stimulation vs. affective and social disclosure), the behaviors they were likely to elicit greatly differed.

The two approaches, ethograms and LMA, were used in concert because they could contribute to the understanding of engagement-related behavior in different ways. On the one hand, ethograms, which are descriptive in nature, could give us the possibility to create a lexicon of engagement-related behavior without selecting the meaningful behaviors *a priori*. On the other hand, LMA, which is a holistic framework to describe and interpret movement, could enable us to find the structure of engagement-related behavior by appealing to the function of each behavior.

##### Development of the ethograms

In order to develop the two ethograms, we first watched the thirty videos allocated to the construction of the coding system (thirty sessions: 15 of game-based cognitive stimulation, 15 of robot-based free play). Then, we described each video in a separate file by detailing the main events and behaviors in chronological order. Further, we watched each video at a slow speed and stopped it whenever we identified a micro-behavior. Dautenhahn and Werry (2002) defined micro-behaviors as well-identifiable low-level action-movement-oriented behaviors recognizable by computational systems. Each micro-behavior was given a name and an operational description.

Before proceeding to the structuring phase, we removed from the ethograms those micro-behaviors that had an ambiguous meaning. For instance, we removed manipulators (e.g., scratching the chin or the scalp), adjustments (e.g., adjusting spectacles, watch, bracelets, earrings, clothes), and vocalizations (e.g., sighing and singing). These micro-behaviors occurred several times with different meanings. Sometimes participants scratched their scalp due to mental effort, other times simply because of itching. Sometimes they adjusted their clothes because of being fidgety, others due to discomfort. Sometimes they sang as a form of enjoyment, for instance while stroking Pleo. Other times, they sang while in an impasse during board games. The lack of a univocal meaning made it hard to figure out the contribution of these micro-behaviors to the assessment of engagement and brought us to their exclusion.

We also excluded verbal behavior from the ethograms. This decision was made with our target group in mind. Although the participants in this study had their language skills still preserved, most people with dementia do not (Thompson, 1987; Klimova and Kuca, 2016) and it is of crucial importance to make meaning of their engagement-related behavior without being dependent on language production.

As a last step, we discarded head gestures, such as nodding, negation, head protruding, and co-speech gestures (i.e., hand and arm movements that accompany spoken language such as saying no with the index finger of the hand). These gestures were hard to relate to a specific element in the activity without resorting to language. For instance, suppose that a participant nods as a reply to the facilitator while s/he (the participant) is looking at the game. The information conveyed by the question of the facilitator would get lost in the analysis of nonverbal behavior and we might wrongly infer the nodding to be referred to the game.

##### Structuring of the ethogram

As a first step in the structuring of the ethograms into a unique coding system, we stated the body portion involvement. We did so by making reference to the micro-behaviors in the two ethograms. In LMA, body portion involvement refers to which body parts are activated during movement (Hackney, 2002). The involvement might regard the whole body or single body parts. A whole body movement is a movement in which the body is involved in its entirety. For instance, walking on the street. A body part movement (also called gesture) is a movement that involves just a discrete part of the body. For instance, a head turning. The body parts involved in a movement might be *body areas* (i.e., head, torso, chest and pelvis), *limbs* (i.e., arms, hands, legs, feet), *joints* (e.g., shoulders, elbow, wrists), and *body quadrants* (i.e., right upper, left upper, right lower, left lower). The micro-behaviors in the ethograms involved two body areas, *head*, and *torso*, and two limbs, *arms*, and *hands*. Given that there were no specific behaviors in the ethograms involving exclusively the arms or the hands, we decided to group the two limbs in a single category. As a result, the body portion involvement of both the ethograms consisted of three body parts: head, torso and arms/hands. We grouped the micro-behaviors in the two ethograms according to the body part they involved.

As a second step of the structuring, we identified those micro-behaviors expressing a directional shape of the body parts (i.e., head, torso, arms/hands) and organized them based on their target in space. From the perspective of one of the participants, we identified five *foci* of the micro-behaviors: the partner, the facilitator, the experimenter, the game (i.e., the board games or Pleo) and none of them. Most micro-behaviors in the two ethograms described the movement of a body part aimed at addressing or physically reaching one of the foci. For instance, *gaze toward the partner* was a *head* movement aimed at addressing the focus *partner*. Similarly, *lean in partner* was a *torso* movement aimed at physically addressing the focus *partner*. Last, *touch the partner* was an *arms/hands* movement aimed at physically reaching the focus *partner*.

As a concluding step in the structuring of the ethograms, we studied the remaining micro-behaviors. We noticed that some of them expressed a directional shape with shaping support, while others a shape flow occurring simultaneously with a directional shape. These micro-behaviors could be seen as traits superimposed on the directional shape carrying an additional item of meaning in terms of engagement. Indeed, they all described an affective attitude of the participant, either positive or negative, toward the foci of the activity. We grouped these micro-behaviors according to their meaning in terms of affect: positive or negative. There was just one exception to this paradigm which regarded micro-behaviors such as applauding or dancing with the arms and hands. These micro-behaviors did not express a directional movement toward the foci of the activity, but exclusively a gestural movement carrying a positive affective meaning.

#### The coding system

The coding system resulting from the structuring process of the two ethograms can be retrieved in Tables A, B, and C in Appendix A (see Supplementary Material). We traced the structure of the ethograms (body portion involvement, directional shape, shape flow and shaping) back to a coding scheme that could be scored using the software Noldus Observer XT 10.5. In this section, we explain how we achieved this.

Observer XT gives the possibility to define clusters of behaviors, also called behavior groups. Behaviors in the groups can be either mutually exclusive (they cannot overlap in time) or start/stop behaviors (they can co-occur). For the former, the coder just needs to specify the start of a behavior and Observer XT assumes that the previous behavior is concluded. For the latter, the coder needs to specify both the beginning (start) and the end (stop) of the behavior, as Observer XT cannot infer it. In our case, we defined three behavior groups corresponding to the body parts involved in the activities: *head behaviors, torso behaviors*, and *arms/hands behaviors*.

The behaviors in each group were those directional shape micro-behaviors that we had organized based on their focus in the activity (partner, experimenter/facilitator, game, and none of them). Also, micro-behaviors such as applauding and dancing with the hands, which we had described as exceptions to our paradigm, were scored as behaviors (see *positive signs of affection involving arms/hands* and *negative signs of affection involving arms/hands*) and nested in the corresponding behavior group (arms/hands behaviors). As they were addressed to different foci, the behaviors in each behavior group did not overlap, thus we scored them as mutually exclusive^3^.

Another feature of Observer XT is the possibility to add modifiers to behaviors in the behavior groups. Modifiers are additional specifications regarding a behavior that describe it more precisely or limit its scope. We chose to score the micro-behaviors expressing affect as modifiers. These micro-behaviors were scored according to their affective meaning, positive or negative. Moreover, we added a neutral meaning which was scored when the directional shape behaviors appeared alone.

### Research questions and hypotheses

The research questions that we wanted to investigate with this study were four: (i) Is the coding system that we have developed reliable across different coders? (ii) Are there any recurring patterns of engagement-related behavior visible from the scoring of the coding system? (iii) Can we compile an evidence-based model using these recurring patterns? And, if yes, (iv) How good is the fit of this model?

### Ethical approval

The study was conducted according to the Declaration of Helsinki, and to Spanish laws number 159/2007 and 41/2002. An informed written consent was signed by all the legal guardians of participants. All participants were informed about the study and gave their consent to participate. Both the consent of the legal guardian and that of the participant were required to take part in the data collection.

## Results

### Inter-rater reliability

Inter-rater reliability (IRR) was performed on 12 videos (29% of the database). The videos were scored by two coders: the researcher involved in the study (GP) and an external independent coder that had not been involved in the study (TvT).

IRR between coders was calculated using the software Noldus Observer XT 10.5 with the Cohen's kappa statistic (Cohen, 1960). Observer XT calculates IRR by taking into account both the matching between the behaviors scored by the two coders and their overlap in time. We computed the global Cohen's kappa of the coding system, the Cohen's kappa of the behavior groups and the Cohen's kappa of single behaviors. With regards to the latter, we included in the calculation of the IRR only behaviors with a mean duration higher that 1% of the session. Indeed, the Cohen's kappa statistic is not accurate with very infrequent behaviors. We report kappa coefficients of behaviors occurring <5% of the time. However, we suggest to interpret them with caution (Dael et al., 2012).

To evaluate the results of IRR, we referred to the thresholds set by Fleiss (1981) and Bakeman and Gottman (1987). Fleiss suggested that a kappa between 0.40 and 0.60 represented a fair agreement, between 0.60 and 0.75 a good agreement and above 0.75 an excellent agreement. Bakeman and Gottman (1987) considered a kappa coefficient lower than 0.70 as insufficient and proposed to interpret it with suspicion.

We report the results of the IRR in Table 2. With regards to the global IRR, this proved to be excellent for the game-based cognitive stimulation (kappa = 0.78) and very good for the robot-based free play (kappa = 0.74). As for behavior groups, IRR was excellent for head behaviors in the game-based cognitive stimulation (kappa = 0.76) and good in the robot-based free play (kappa = 0.70), very good for torso behaviors in both game-based cognitive stimulation (kappa = 0.74) and robot-based free play (kappa = 0.73) and good for arms/hands behaviors in game-based cognitive stimulation (kappa = 0.63), and robot-based free play (kappa = 0.71).

**Table 2:** Inter-rater reliability with a tolerance window of 3 s.

	**COGNITIVE GAMES**					**ROBOT PLAY**				
**Behavior**	**Duration (%)**			**Reliability**		**Duration (%)**			**Reliability**	
	**Mean**	**Min**	**Max**	**Prop**.	**Kappa**	**Mean**	**Min**	**Max**	**Prop**.	**Kappa**
GP	2.19	0.24	5.60	0.83	0.70	13.70	3.16	22.41	0.82	0.69
GFE	5.31	1.79	12.24	0.83	0.71	4.48	0.42	12.29	0.75	0.59
GG	88.46	81.86	92.19	0.87	0.78	73.30	59.26	96.16	0.81	0.70
NoneH	4.04	2.27	6.65	0.76	0.59	8.52	0.27	15.69	0.67	0.46
**HEAD**	**100**			**0.81**	**0.76**	**100**			**0.76**	**0.70**
LIP	0.03	0.00	0.23	–	–	0.42	0.00	3.48	–	–
NRLTG	58.96	10.58	100.00	0.87	0.76	41.67	6.80	100.00	0.81	0.71
NoneT	41.01	0.00	89.43	0.87	0.75	57.91	0.00	93.21	0.89	0.79
**TORSO**	**100**			**0.87**	**0.74**	**100**			**0.82**	**0.73**
RoP	0.67	0.00	2.89	–	–	0.41	0.00	1.31	–	–
RoFE	0.06	0.00	0.43	–	–	0.11	0.00	0.32	–	–
MG	64.13	49.94	75.97	0.77	0.59	45.03	6.49	84.33	0.80	0.70
SOApos	0.16	0.00	1.57	–	–	0.18	0.00	1.72	–	–
NoneAH	34.98	23.94	47.18	0.82	0.67	54.27	15.09	92.21	0.88	0.77
**ARMS/HANDS**	**100**			**0.77**	**0.63**	**100**			**0.79**	**0.71**
**ELISCE**	**100**			**0.80**	**0.78**	**100**			**0.77**	**0.74**

*(–) The duration of the behavior is too short to allow interpretation. The results of inter-rater reliability for the behavior negative signs of affection of the torso (SOAneg) are not reported as they did not occur in the 12 videos*.

With regards to single behaviors, IRR was good to excellent for most of them. However, for some of them it could not be scored due to a frequency issue (<1%) and for few of them it achieved unsatisfying results. We explained the unsatisfying kappa coefficients of “*none of the target head movements*” in the game-based cognitive stimulation and of “*gaze toward facilitator/experimenter*” in the robot-based free play with their low occurrence (<5%). With regards to the low agreement of “*none of the target head movements*” during robot-based free play, it could be due to the sharp differences in the frequency of the behavior across participants (from 0.27 to 15.69%). For what regards “*manipulate the game*” in cognitive games, the moderate agreement could not be justified by appealing to a frequency issue. In this case, the disagreement was due to an incorrect classification of the behavior by the coders.

### The building of the evidence-based model of engagement-related behavior

In this section, we describe how LMA enabled us to identify patterns of engagement-related behavior, associate a meaning in terms of engagement to them and organize them in a model.

#### The organization of body parts

**Figure 2:**
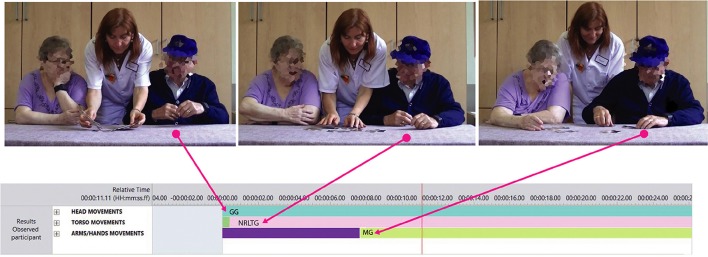
Main pattern of body part organization.

In order to identify patterns of behaviors from the scoring of the coding system, we made reference to the formalization of body part organization (Hackney, 2002). Across activities, we observed a main pattern of body part organization in the directional shape behaviors: *successive*—*space hold* (with various gestures)—*successive* (see Figure 2). Successive organization appeared after the game was placed on the table by the facilitator. In this situation, the movement toward the game was initiated by the head and sequenced into arms/hands via the torso. After this successive movement, the head and the torso remained in the same position, while the arms/hands kept manipulating the game. This last organization of body parts can be described as a *space hold* of the head and torso with gestures of arms/hands. In LMA, space hold is the lock of specific body parts in space. When the game was removed from the table by the facilitator, we observed yet another instance of successive organization. The head of the participant initiated the movement of the torso backwards toward the seat. Then, this movement toward the seat progressed through the torso into the arms/hands. The pattern of body part organization *successive*—*space hold* (with various gestures)—*successive* occurred also when the participant wanted to address the partner. In this case, however, the initiation of the head led into a sideways movement of the torso toward the partner which then progressed into arms/hands. From the described patterns of body part organization, we hypothesized that movements directed toward the game and the partner were initiated by the head and that the head had a leading role in engagement-related behavior.

**Figure 3:**
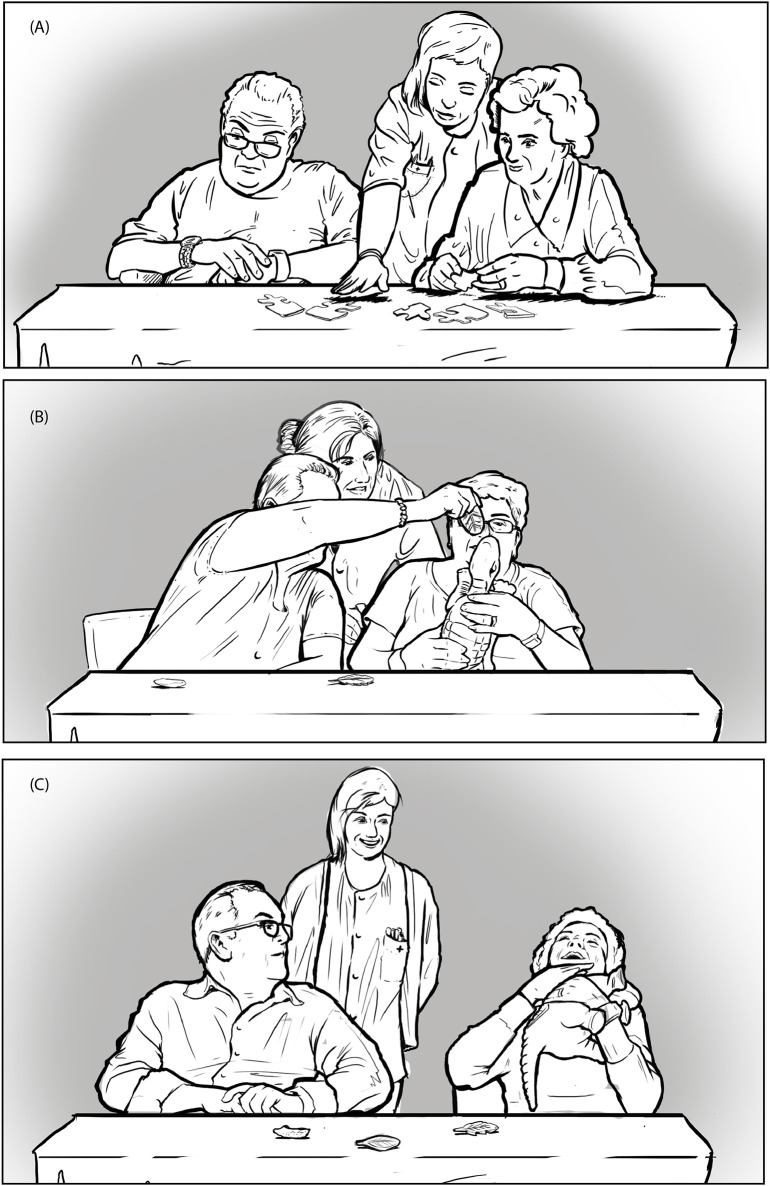
**(A)** *Man:* no body part is addressed toward the game or the partner (no engagement). *Woman:* all body parts are simultaneously addressed toward the game (full active participation); **(B)** *Woman on the left:* all body parts are simultaneously directed toward the partner (full active social engagement). *Woman on the right:* all body parts are directed toward the game (full active participation). **(C)** *Man*: just the head is directed toward the partner (social acknowledgement). Woman: all body parts are directed toward the game. Moreover all body parts express positive affect (positive full active participation).

The *successive*—*space hold* (with various gestures)—*successive* pattern of body part organization was the most frequent during activities and, as it involved all the body parts specified in the ethograms, we called it *full active participation* when it was directed toward the game (see the woman in Figure 3A and the woman on the right in Figure 3B) and *full active social engagement* when it was directed toward the partner (see the woman on the left in Figure 3B).

**Figure 4:**
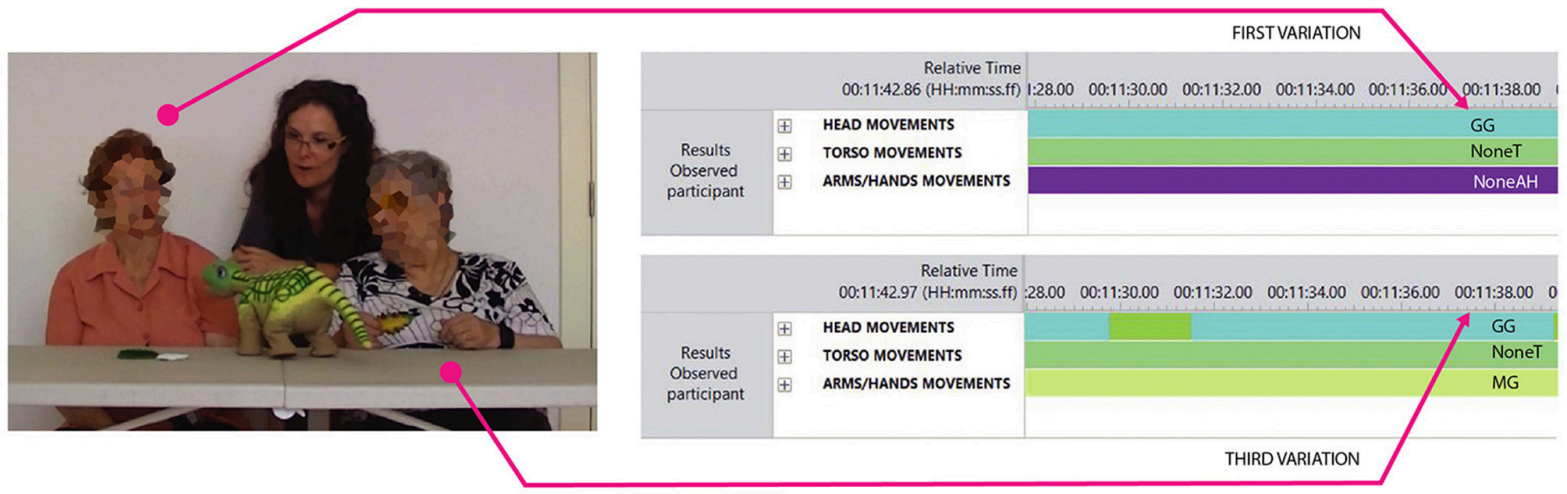
Examples of the first and third variations to the main pattern of body part organization.

The *successive*—*space hold* (with various gestures)—*successive* pattern of body part organization did not always involve all the body parts specified in the ethograms. We observed three *variations* of the main pattern of body part organization. The first occurred when the participants addressed the game (or the partner) exclusively with the head and held the head in the same position in space (see Figure 4). The second appeared when they addressed the game (or the partner) with a successive movement of the head and the torso which was then held in the same position in space. The third took place when the participants addressed the game (or the partner) with a sequential movement of the head and the arms/hands which was then held in the same position in space, without further activation of the torso (see Figure 4).

Following Judge et al. (2000), we considered the first two variations as passive forms of engagement, as they did not involve a proactive manipulation of the artifacts in the activity. However, we acknowledged that the second variation was a step forward in terms of engagement with respect to the first one, as it also involved the activation of the torso. As a matter of fact, postural attitudes express a “corporeal readiness to act” (Bull, 1951; Sheets-Johnstone, 1999). With regards to the last variation, it did describe a constructive form of engagement. Nonetheless, the lack of torso involvement made this engagement look less complete. We called the first variation *passive attention* when the head was directed toward the game and *social acknowledgment* when it was directed toward the partner (see the man in Figure 3C). We called the second variation *attentional readiness* when the head and torso were directed toward the game and *social readiness* when they were directed toward the partner. We called the third variation *reduced active participation* when the head and the arms/hands were directed toward the game and *reduced active social engagement* when they were directed toward the partner. As a consequence of this reasoning, we can conclude that: the more body parts are involved in the movement toward the game or toward the partner, the higher the engagement of the participant.

#### The organization of modifiers

On top of these more directional movements, the coding system also featured behaviors expressing positive and negative affect. As these behaviors were superimposed on the directional ones, they added a further layer of meaning to them. The affective coloring specified the valence of engagement.

As affective behaviors vary due to the affordances and the uses of the artifacts in the activity, their patterns of body part organization greatly depend on the type of activity. For instance, during game-based cognitive stimulation, we could not isolate specific patterns of body part organization as affective behaviors were very rare. What we noticed, however, was that affective behaviors mostly appeared after the completion of the game and mostly involved the head (e.g., smile and laughter). As for robot-based free play, affective behaviors were rather frequent and involved two types of body part organization: *successive and space hold (with or without various gestures)*. A typical successive body part organization appeared when the participant directed the head toward Pleo, smiled at it, initiated the approach toward Pleo with the chest, embraced the robot with both arms/hands, lift the robot to bring it close to the torso and hugged it. This sequence was sometimes followed by a space hold of the three body parts in the affective behavior (see the woman in Figure 3C). Other times, it was followed by a space hold of the two body parts with gestures (e.g., hug the robot while stroking it, hug the robot and cradle it). Another successive body part phrasing occurred when the participant addressed Pleo with the head, smiled at it, initiated the approach toward Pleo with the chest and stroked the robot. Also in this case, the sequence was often followed by a space hold with gestures. This brought us to conclude that the more body parts are involved in the expression of affect, the more intense is the affective coloring of engagement.

### The model

To sum up, the analysis of body part organization brought us to the following conclusions:

1. The head has a leading role in engagement-related behavior.
2. The head initiates the movement of the torso toward the activity (i.e., the game or the partner).
3. The movement of the torso toward the activity can be then sequenced into arms/hands.
4. The head alone might initiate the movement of the arms/hands toward the activity.
5. The gestural support (positive or negative) might initiate the postural support (positive or negative).
6. The postural support (positive or negative) can be then sequenced into quality of gesture (positive or negative).
7. The gestural support (positive or negative) alone might initiate the quality of gesture (positive or negative).

These conclusions were transformed into seven hypothetical relations between variables^4^. Respectively:

(H1) The variable *gaze toward activity* (i.e., head directed toward the game and the partner) is an exogenous variable (i.e., a variable whose value is not dependent on the value of other variables in the model).
(H2) The variable *gaze toward activity* (i.e., head directed toward the game and the partner) has a direct effect on the variable *lean toward activity* (i.e., torso directed toward the game and the partner).
(H3) The variable *lean toward activity* has a direct effect on the variable *reach out activity* (i.e., arms/hands reaching the game and the partner).
(H4) The variable *gaze toward activity* (i.e., head directed toward the game and the partner) has a direct effect on the variable *reach out activity*.
(H5) The variable *gaze toward activity with gestural support* (i.e., affective behaviors involving the head directed toward the game and the partner) has a direct effect on the variable *lean toward activity with postural support* (i.e., affective behaviors involving the torso directed toward the game and the partner).
(H6) The variable *lean toward activity with postural support* has a direct effect on the variable *reach out activity with quality of gesture* (i.e., affective behaviors involving the arms/hands directed toward the game and the partner).
(H7) The variable *gaze toward activity with gestural support* has a direct effect on the variable *reach out activity with quality of gesture*.

On top of these seven hypothetical relations, we added three additional ones. These were aimed at disclosing relationships between the behaviors and the modifiers pertaining to the same body part (i.e., head, torso, arms/hands).

(H8) The variable *gaze toward activity* has a direct effect on the variable *gaze toward activity with gestural support*.
(H9) The variable *lean toward activity* has a direct effect on the variable *lean toward activity with postural support*.
(H10) The variable *reach out activity* has a direct effect on the variable *reach out activity with quality of gesture*.

**Figure 5:**
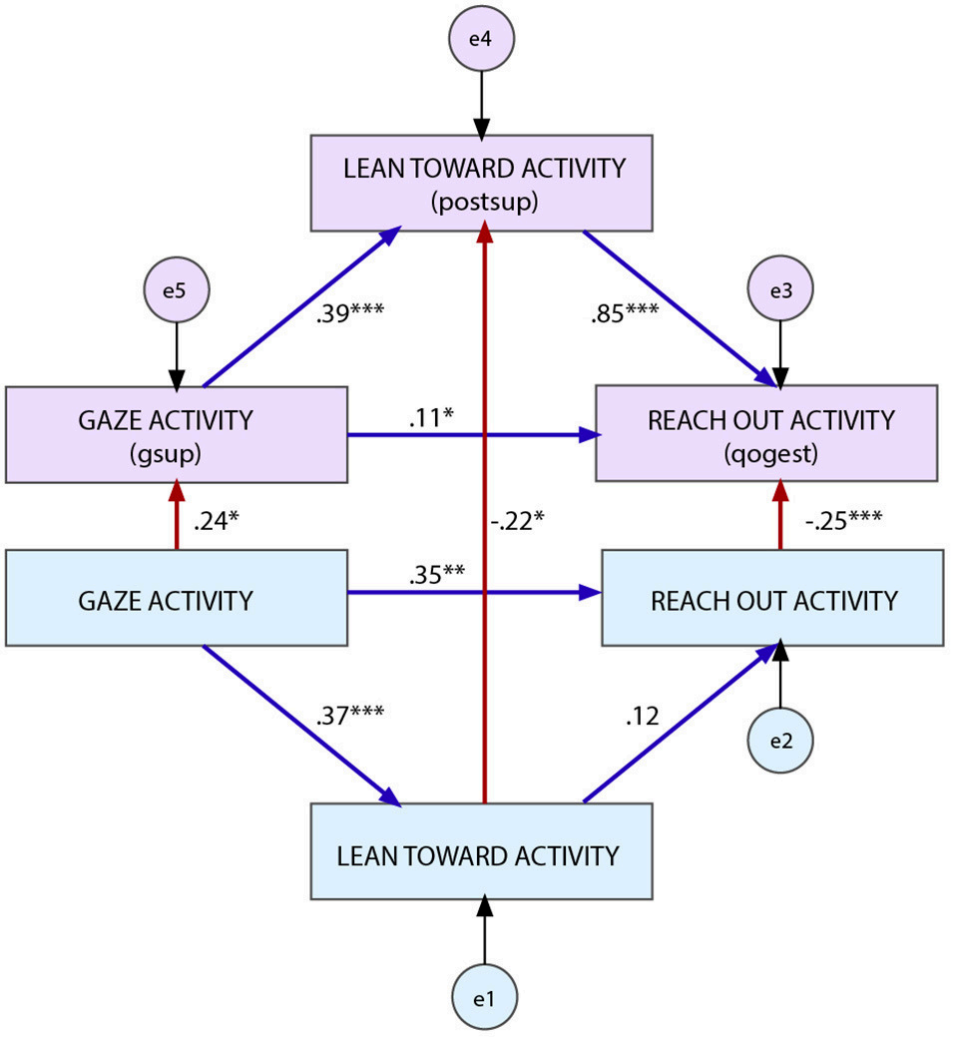
SEM of the evidence-bades model of engagement-related behavior. Significance level: ^*^p < 0.05, ^**^p < 0.01, ^***^p < 0.001.

The model in Figure 5 depicts all the hypothetical relationships between variables (H2–H7: blue arrows, H8–H10: red arrows).

### Test of the evidence-based model of engagement-related behavior

To test the model, we scored all 42 videos in the database with the coding system and calculated the percentage of observation duration of each behavior and modifier. Before fitting the model, we performed a series of operations to reduce the data. To preserve the clarity of this section, we detail them in Table 3. The rationale behind the data reduction was suggested by our model. Indeed, we treated those behaviors directed toward the game or the partner as *engagement-related behaviors* and those behaviors directed toward the facilitator/experimenter or not directed toward the foci of the activity as *disengagement-related behaviors*. The result of the data reduction was an engagement score for each body part ranging from −100 to 100, where −100 represented the highest disengagement with the activity (game and partner) and 100 the highest engagement with it. With regards to the modifiers, we took into account only the positive and negative modifiers referred to the game and the partner. We subtracted the negative modifiers from the positive. Thus, we obtained a negative score when negative engagement was predominant, a positive score when positive engagement prevailed and a score of zero when negative and positive engagement were even.

**Table 3:** Data Reduction for SEM.

**Variable name**	**Abbreviation**	**Data reduction**
GAZE ACTIVITY	GAct	= (GP + GG) - (GFE + NoneH)
LEAN TOWARD ACTIVITY	LTAct	= (LIP + NRLTG) – (NoneT)
REACH OUT ACTIVITY	RoAct	= (RoP + MG) – (RoFE + NoneAH)
GAZE ACTIVITY (gestural support)	GAct_gsup	= (GP_pos + GG_pos) – (GP_neg + GG_neg)
LEAN TOWARD ACTIVITY (postural support)	LTAct_postsup	= (LIP_pos + NRLTG_pos) – (LIP_neg + NRLTG_neg)
REACH OUT ACTIVITY (quality of gesture)	RoAct_qogest	= (RoP_pos + MG_pos + SOA_pos) – (RoP_neg + MG_neg + SOA_neg)

We tested the model using SEM with the software SPSS Amos 22.0. We ran the model twice using the data from both activities. The first time, we calculated the Mahalanobis distance and identified the farthest observations from the centroid ones. The second time we fitted the model excluding the outlier observations (*N* = 7). Indeed, SEM is sensitive to violations to normal distribution. The model proved to be an excellent fit for the data [*X*^2^_(6, N = 77)_ = 5.866, *p* = 0.436; *RMSEA* = 0.000; *NFI* = 0.970; *CFI* = 1.000; *RFI* = 0.896; *PNFI* = 0.277] and almost all the hypothesized relations (H1–H10) between variables were significant (see Table 4 and Figure 5). H1 was confirmed by the goodness of fit of the model. H2–H10 were confirmed both by the goodness of fit of the model and by the significance of the model estimates. The only postulated relation between variables that was not significant was the one between *lean toward the activity* and *reach out activity* (H3). We ran two regression analyses to figure out whether this result depended on the behaviors directed toward the game or on those directed toward the partner. The results disclosed that *near reach/lean toward game* had a significant effect on *manipulate game* (β = 0.246, *t*_(76)_ = 2.201, *p* < 0.05) and *lean in partner* had a significant effect on *reach out partner* (β = 0.231, *t*_(76)_ = 2.057, *p* < 0.05). Compared to regression analysis, SEM calculates also an error term for the variables. Thus, the lack of a significant result for this relation depended on the size of the error term of the two variables and not on the lack of relationship between them.

**Table 4:** Path estimates of the evidence-based model of engagement-related behavior.

	**Hypothesized path**	**Estimate**	**S.E**.	**C.R**.	***p***	**Hypothesis supported yes/no**
H2	GAct → LTAct	0.372	0.680	3.499	^***^<0.001	Yes
H3	LTAct → RoAct	0.122	0.060	1.079	>0.05	No
H4	GAct → RoAct	0.349	0.386	3.099	^**^<0.01	Yes
H5	GAct → GAct_gsup	0.240	0.099	2.159	^*^<0.05	Yes
H6	RoAct → RoAct_qogest	−0.255	0.017	−5.998	^***^<0.001	Yes
H7	GAct_gsup → LTAct_postsup	0.390	0.106	3.750	^***^<0.001	Yes
H8	LTAct_postsup → RoAct_qogest	0.845	0.068	18.563	^***^<0.001	Yes
H9	GAct_gsup → RoAct_qogest	0.112	0.069	2.444	^*^<0.05	Yes
H10	LTAct → LTAct_postsup	−0.218	0.015	−2.102	^*^<0.05	Yes

Significance level:

* *p < 0.05*,

** *p < 0.01*,

*** *p < 0.001*.

With regards to the negative relations between the behavior *lean toward activity* and the modified *lean toward activity with postural support* and between the behavior *reach out activity* and the modified *reach out activity with quality of gesture*, these might be due to the fact that *lean toward activity* and *reach out activity* were more frequent during game-based cognitive stimulation, whereas their modified subsets *lean toward activity with postural support* and *reach out activity with quality of gesture* were more frequent during robot-based free play. Hence, the former could not be positive predictors of the latter in both activities.

### Limitations

The present study has been mainly limited by the small sample size. Future work should attempt to increase the sample size and include people with dementia coming from different cultural backgrounds and countries. Moreover, it should test whether the model holds in activities others than game-based cognitive stimulation and robot-based free play and in activities carried out in larger groups.

A further aspect to study is related to the set-up of the cameras. The second coder has reported that the chosen set-up of the cameras made it complex to score postural shifts. The frontal camera flattened the view and the other camera was not lateral enough to catch the detaching of the torso of the participant from the seat. We suggest that a good set-up to collect data should consist of three cameras, one frontal and two lateral and that the lateral cameras should be positioned exactly on the side of participants. This set-up would not just help in properly scoring postures, but also in scoring arms/hands movements occluded by objects.

Another limitation of this study is the number of coders. We tested the agreement of the coding system with two coders. However, a larger number of annotators would have made the analysis of IRR stronger. For what concerns statistical analyses, SEM is a statistic usually performed on large samples. In order to perform it, we used the scores of the same participants in different sessions as different observations. However, a better practice would be that of having a larger sample size and using just one score per participant.

## Discussion

The body of work described in the present paper significantly advances the state of the art. We developed a coding system aimed at measuring engagement-related behavior across activities in people with dementia and a model to interpret the results of such a coding system in terms of engagement. We call the coding system the Ethographic and Laban-Inspired Coding System of Engagement (ELICSE) and the model the Evidence-based Model of Engagement-related behavior (EMODEB). Both have been developed for people with dementia. However, their use can be extended to people with difficulty of introspection and verbal communication (e.g., persons with autism).

### Discussion on the coding system

With respect to observational rating scales, the ELICSE enables researchers to study the behavior of people with dementia in its complexity and temporal progression. Compared to available coding schemes, it gives an account of the continuous flow of behavior. As opposed to ethograms, it helps researchers to trace behavior back to an overall engagement state. The ELICSE achieved an excellent IRR in both game-based cognitive stimulation and robot-based free play (first research question: positive response).

The ELICSE has been developed in the context of game-based cognitive stimulation and robot-based free play. However, for its characteristics, it can be applied to activities that: (i) do not entail physical effort, (ii) envisage a proactive role for the person with dementia, and (iii) involve the use of tangible artifacts (e.g., social robots, sensory stimulation, interactive technologies).

In order to apply the ELICSE to other activities, the researcher should adapt it. In any activity, there is a different context and a different body portion involvement. The context refers to the objects and actors in the activity (i.e., facilitator, experimenter, participant, robot, jigsaw puzzle, relative). The body portion involvement refers to the body parts involved in the movement toward the objects and the actors in the activity and in the expression of affect. The behaviors in the ELICSE can be generated by combining the body parts involved in the activity with the actors and objects in it. The modifiers in the ELICSE arise from the positive and negative behaviors superimposed on the more directional ones.

For instance, suppose we would like to measure engagement in a group-based sensory stimulation. We know that the sensory stimulation activity is carried out by two facilitators in groups of six people and that it features the use of patches with different textures. We also know that the sensorial stimulation is carried out in a sitting position and participants sit all around a circular table. The actors and objects of our activity would be three in this context: the *facilitators*, the *participants*, and the *patches*. As participants are sitting during the activity, the body parts addressing the facilitators, the other participants and the patches are likely to be the *head*, the *torso* and the *arms* and *hands*. The behaviors in the ELICSE would be the movements of the head, the torso and the arms/hands toward the facilitators, the other participants or the patches. The modifiers would be the behaviors superimposed on the directional ones expressing positive or negative affect. As an example, the movement of the head toward the patches would be *gaze toward the patches*, while the movement of the torso toward another participant would be *lean toward/in the other participant*.

### Discussion on the model

The EMODEB is a model that describes the natural flow of behavior across body parts and details the meaning of different patterns of body part organization appearing during activities in terms of engagement. In agreement with the framework of Castellano and colleagues, the ELICSE is composed of three elements: task-engagement, social interaction and affect.

In concordance with Sidner et al. and O'Brien and Toms, the EMODEB identifies three stages in the expression of engagement, which are described in terms of patterns of body part organization (second research question: positive response). The *successive movement* of participants toward the game or the partner might be assimilated to the start of the engagement (or *point of engagement*) in the activity. The *shape hold (with various gestures)* can be interpreted as the maintenance of engagement (or *sustained engagement*). The *successive movement* of participants back to the seat might be read as the end of engagement (or *disengagement*). The relation between the body parts involved in the movement were summarized in a structural equation model (third research question: positive response). The SEM achieved an excellent fit and the relations between the body parts involved in the movement were all significant (forth research question: positive response).

According to the EMODEB, the behaviors in the ELICSE do not have the same importance in engagement terms as they are organized hierarchically. Based on the EMODEB, *gaze toward activity* is more important than *lean toward activity* and *reach out activity*, and *reach out activity* is more important than *lean toward activity*. Likewise for modifiers, *gaze toward activity with gestural support* is more important than *lean toward activity with postural support* and *reach out activity with quality of gesture*, and *reach out activity with quality of gesture* is more important than *lean toward activity with postural support*. The EMODEB demonstrates that the head behaviors and the arms/hands behaviors are respectively the starting and the conclusive point of both engagement-related (*gaze toward activity, lean toward activity, reach out activity*) and affect-related behavior (*gaze toward activity with gestural support, lean toward activity with postural support, reach out activity with quality of gesture*), and that the torso behaviors energize the passage between the start and the end of engagement-related and affect-related behavior without playing a substantial role in it.

The hierarchical organization of the behaviors in the ELICSE also supports our statement about the four levels of intensity of engagement-related behavior: (i) *passive engagemen*t (passive attention and social acknowledgement): head directed toward the game or the partner, (ii) *readiness to engage* (attentional readiness and social readiness): head and torso directed toward the game or the partner, (iii) *reduced active engagement* (reduced active participation and reduced active social engagement): head and arms/hands directed toward the game or the partner without the involvement of the torso (iv) *full active engagement* (full active participation and full active social engagement): head, torso and arms/hands directed toward the game or the partner. Moreover, it also backs up the existence of a similar hierarchical structuring for affective behaviors.

The four levels of engagement-related behavior are an expression of what Brown and Cairns defined as the first level of immersion in a game (the gamer invests time, effort and attention). The affective coloring of engagement in the EMODEB can have a negative or positive valence. Its intensity depends on the number of body parts involved in expressing affect. The affective coloring of full active engagement can be compared to the second level of immersion in a game (the gamer's emotions are directly affected by the game). As expected, the third level of immersion in the game formalized by Brown and Cairns, could not be isolated in the behavior of people with dementia during activities.

Future work on the EMODEB should focus on getting overall scores of engagement and affect and on scoring the intensity of engagement over time. The former goal can be achieved by assigning weights to each body part based on their importance in the model (e.g., head = 0.50, torso = 0.10, and arms/hands = 0.40) and computing weighted averages of engagement and affect. The latter objective could be fulfilled by associating a score to each level of engagement (e.g., from 1 to 4, where each unit is a level of engagement) and affect (e.g., − 3 to +3, where −3 is negative engagement expressed with all three body parts and +3 positive engagement expressed with all three body parts) and code the progression of engagement over the session with a time-sampling technique. To the best of our knowledge, the EMODEB constitutes the first formalization of the way the engagement-related behavior of people with dementia naturally occurs and unfolds.

## Conclusions

The present paper reported the results of a study aimed at developing a coding system of engagement-related behavior and a model of engagement-related behavior for people with dementia. The first objective resulted in the ELICSE, a coding system that can be used to quantify engagement-related behavior across diverse activities, but also to describe how this changes over time. The second objective led to the EMODEB, an evidence-based model of engagement that describes relationships between the behaviors of different body parts and associates them a meaning in terms of engagement. The ELICSE achieved an excellent IRR and the EMODEB proved to be an excellent fit for our data. The ELICSE and the EMODEB were developed in the context of game-based cognitive stimulation and robot-based free play. However, their use can be extended to other activities. To the best of our knowledge, the ELICSE and the EMODEB constitute the first formalization of engagement-related behavior for dementia that describes how behavior unfolds over time and what it means in terms of engagement.

## Author contributions

GP contributed to the conception, design, data collection, analysis and interpretation of the study; RvB contributed to the analysis and interpretation of the study; MD-B, AC-M, EB, and MR supervised the conception, design, data collection, analysis and interpretation of the study; GP wrote the paper; RvB contributed to the writing and critical revision of the paper; MD-B, AC-M, MR, and EB: critically reviewed the paper and gave the final approval of the version submitted.

### Conflict of interest statement

The authors declare that the research was conducted in the absence of any commercial or financial relationships that could be construed as a potential conflict of interest.

## Footnotes

^1^Note that we used the term coding system, instead of coding scheme. A *coding scheme* is a portion of an ethogram. A *coding system* is an organization of the behaviors in the ethogram.

^2^As the LMA framework provides a holistic approach to movement, we would like to emphasize that the focus on the category shape did not entail an abandon of the other categories.

^3^As a side note, we would like to inform the reader that from now on we will use the term *micro-behaviors* to refer to the low-level action-movement-oriented behaviors in the ethograms. Instead, we will speak about *behaviors* to refer to the clusters of micro-behaviors in the coding system.

^4^From this moment of the paper on we will use the term *variable* instead of behavior and we will use the variables' names detailed in Table 3.
