# Supplementary material for: Understanding Engagement in Dementia Through Behavior. The Ethographic and Laban-Inspired Coding System of Engagement (ELICSE) and the Evidence-Based Model of Engagement-Related Behavior (EMODEB)
Source: Front Psychol. 2018 May 24;9:690. doi: 10.3389/fpsyg.2018.00690 (PMC5976786; doi:10.3389/fpsyg.2018.00690)
Supplement: Supplementary file 1 [file Data_Sheet_1.pdf]

frontiers

Appendix A –The Coding System

HEAD BEHAVIORS (directional)	MODIFIERS (affective) – gestural support
**Gaze toward partner**<br>The observed participant*[1] directs the head and the eyes or just the eyes toward the partner*[2]<br><br>**CODE: GP**	***positive* gestural support (code: GP_pos)**<br>*with* facial expressions or gestures that display positive affect: smile, laugh, stick the tongue out, send/give kisses<br><br>***no* gestural support (code: GP_no)**<br>without facial expressions or gestures that display positive affect<br><br>***negative* gestural support (code: GP_neg)**<br>*with* facial expressions or gestures that display negative affect: anger, sadness, disgust, fear, frowning, boredom (yawn), pain
**Gaze toward facilitator/experimenter**<br>The observed participant directs the head and the eyes or just the eyes toward the facilitator or the experimenter<br><br>**CODE: GFE**	***positive* gestural support (code: GFE_pos)**<br>*with* facial expressions or gestures that display positive affect: smile, laugh, stick the tongue out, send/give kisses<br><br>***no* gestural support (code: GFE_no)**<br>without facial expressions or gestures that display positive affect<br><br>***negative* gestural support (code: GFE_neg)**<br>*with* facial expressions or gestures that display negative affect: anger, sadness, disgust, fear, frowning, boredom (yawn), pain
**Gaze toward game**<br>The observed participant directs the head and the eyes or just the eyes toward the game*[3]<br><br>**CODE: GG**	***positive* gestural support (code: GG_pos)**<br>*with* facial expressions or gestures that display positive affect: smile, laugh, stick the tongue out, send/give kisses, nuzzle the robot, blow of the surface of the robot<br><br>***no* gestural support (code: GG_no)**<br>without facial expressions or gestures that display positive affect<br><br>***negative* gestural support (code: GG_neg)**<br>*with* facial expressions or gestures that display negative affect: anger, sadness, disgust, fear, frowning, boredom (yawn), pain
**None of the target head movements**<br>The observed participants does not perform any of the target head behaviors (see above)<br><br>**CODE: NoneH**	***positive* gestural support (code: NoneH_pos)**<br>*with* facial expressions or gestures that display positive affect: smile, laugh, stick the tongue out, send/give kisses<br><br>***no* gestural support (code: NoneH_no)**<br>without facial expressions or gestures that display positive affect<br><br>***negative* gestural support (code: NoneH_neg)**<br>*with* facial expressions or gestures that display negative affect: anger, sadness, disgust, fear, frowning, boredom (yawn), pain, avoidance, closed eyes

Table A. The ELICSE, description of head behaviors

*[1]*Observed participant*: the observed participant is the one whose behavior we are scoring.

*[2]*Partner*: the partner is the participant who is taking part in the activity with the observed participant, but whose behavior we are not currently scoring.

*[3]*Game*: the game is the physical set of tools that the participants manipulate during the activity. For instance, in dominoes, the tiles of the domino, in puzzles, the pieces of the puzzle, in the robot set, the robot itself, but also its accessories (e.g., leaves, stones).

TORSO BEHAVIORS (directional)	MODIFIERS (affective) – postural support
**Lean in partner**<br>The observed participant leans into the partner by rotating the torso and advancing it in the direction of the partner or by spreading it sideways in the direction of the partner<br><br>**CODE: LIP**	***positive* postural support (code: LIP_pos)**<br>*with* movements of the torso that display positive affect: hug the partner<br><br>***no* postural support (code: LIP_no)**<br>without movements of the torso that display positive affect<br><br>***negative postural support* (code: LIP_neg)**<br>*with* movements of the torso that display negative affect: pull away the partner when s/he is approaching
**Near reach/lean toward the game**<br>The observed participant tilts the torso toward the game or holds the game close to its torso<br><br>**CODE: NRLTG**	***positive* postural support (code: NRLTG_pos)**<br>*with* movements of the torso that display positive affect: hug the robot, cradle the robot, make the robot sleep on the shoulder<br><br>***no* postural support (code: NRLTG_no)**<br>without movements of the torso that display positive affect<br><br>***negative* postural support (code: NRLTG_ neg)**<br>*with* movements of the torso that display negative affect: reject the game by pulling it away
**None of the target torso movements**<br>The observed participants does not perform any of the target torso behaviors (see above)<br><br>**CODE: NoneT**	**(none)**

Table B. The ELICSE, description of torso behaviors

ARMS/HANDS BEHAVIORS (directional)	MODIFIERS (affective) – quality of gesture
**Reach out partner**<br>The observed participant touches or indicates the partner, passes the game to or receives the game from the partner, manipulates the game while this is held by the partner<br><br>**CODE: RoP**	***positive* quality of gesture**<br>The observed participant strokes, pats or hugs the partner. The observed participant waves or strokes the robot, puts the finger in the mouth or takes the chin of the robot, strokes the robot with one of its accessories (i.e., leaves, stones) *when the robot is held by the partner*<br><br>***no* quality of gesture**<br>(See *reach out partner*)<br><br>***negative* quality of gesture**<br>The observed participant rejects or hits the partner. The observed participant rejects the game, hits or puts a finger in the eyes of the robot *when the robot is held by the partner*
**Reach out facilitator/experimenter**<br>The observed participant touches or indicates the facilitator/experimenter, passes the game to or receives the game from the facilitator, manipulates the game while this is held by the facilitator<br><br>**CODE: RoFE**	***positive* quality of gesture**<br>The observed participant strokes, pats or hugs the facilitator. The observed participant waves or strokes the robot, puts the finger in the mouth or takes the chin of the robot, strokes the robot with one of its accessories (i.e., leaves, stones) *when the robot is held by the facilitator*<br><br>***no* quality of gesture**<br>(See *reach out facilitator/experimenter*)<br><br>***negative* quality of gesture**<br>The observed participant rejects or hits the facilitator. The observed participant rejects the game, hits or puts a finger in the eyes of the robot *when the robot is held by the facilitator*
**Manipulate game**<br>The observed participant holds, touches, interacts (e.g., puts the pieces of the puzzle in place, feeds the robot), or indicates the game<br><br>**CODE: MG**	***positive* quality of gesture**<br>The observed participant waves, hugs, cradles or strokes the robot, puts the finger in the mouth or takes the chin of the robot, strokes the robot with one of its accessories (i.e., leaves, stones). The observed participant touches strongly the pieces of the shape or jigsaw puzzle with his/her after combining them or flips the tile of the domino on its center<br><br>***no* quality of gesture**<br>(See *manipulate the game*)<br><br>***negative* quality of gesture**<br>The observed participant rejects, hits or throws away the game or makes the robot fall, turns it upside down and puts a finger its eyes
**Positive signs of affection involving arms/hands**<br><br>The observed participant claps his/her hands, dances, mimics a torero, rhythmically bangs his/her fists on the table<br><br>**CODE: SOA_pos**	**(none)**

**Negative signs of affection involving arms/hands**<br>The observed participant hides its face in the hands, covers its face with the hands or performs insulting gestures<br><br>**CODE: SOA_neg**	**(none)**
**None of the target arms/hands movements**<br>The observed participants does not perform any of the target arms/hands behaviors (see above)<br><br>**CODE: None_AH**	**(none)**

Table C. The ELICSE, description of arms/hands behaviors
